# Levosimendan versus dobutamine for sepsis-induced cardiac dysfunction: a systematic review and meta-analysis

**DOI:** 10.1038/s41598-021-99716-9

**Published:** 2021-10-13

**Authors:** Dong-Hua Liu, Yi-Le Ning, Yan-Yan Lei, Jing Chen, Yan-Yan Liu, Xin-Feng Lin, Zhong-Qi Yang, Shao-Xiang Xian, Wei-Tao Chen

**Affiliations:** 1 Department of Critical Care Medicine, The First Affiliated Hospital, Guangzhou University of Chinese Medicine, No. 16, Jichang Road, Baiyun District, Guangzhou, 510405 Guangdong Province China; 2 The First Clinical School, Guangzhou University of Chinese Medicine, Guangzhou, 510405 China; 3 Ling-Nan Medical Research Center, Guangzhou University of Chinese Medicine, No. 12, Jichang Road, Baiyun District, Guangzhou, 510405 Guangdong Province China; 4 The First Affiliated Hospital, Guangzhou University of Chinese Medicine, No. 16, Jichang Road, Baiyun District, Guangzhou, 510405 Guangdong Province China

**Keywords:** Cardiology, Diseases, Medical research

## Abstract

Levosimendan and dobutamine are extensively used to treat sepsis-associated cardiovascular failure in ICU. Nevertheless, the role and mechanism of levosimendan in patients with sepsis-induced cardiomyopathy remains unclear. Moreover, previous studies on whether levosimendan is superior to dobutamine are still controversial. More importantly, these studies did not take changes (before-after comparison to the baseline) in quantitative parameters such as ejection fraction into account with the baseline level. Here, we aimed to determine the pros and cons of the two medicines by assessing the changes in cardiac function and blood lactate, mortality, with the standardized mean difference used as a summary statistic. Relevant studies were obtained by a thorough and disciplined literature search in several notable academic databases, including Google Scholar, PubMed, Cochrane Library and Embase until November 2020. Outcomes included changes in cardiac function, lactic acid, mortality and length of hospital stay. A total of 6 randomized controlled trials were included in this study, including 192 patients. Compared with dobutamine, patients treated with levosimendan had a greater improvement of cardiac index (ΔCI) (random effects, SMD = 0.90 [0.20,1.60]; I^2^ = 76%, *P* < 0.01) and left ventricular stroke work index (ΔLVSWI) (random effects, SMD = 1.56 [0.90,2.21]; I^2^ = 65%, *P* = 0.04), a significant decrease of blood lactate (Δblood lactate) (random effects, MD =  − 0.79 [− 1.33, − 0.25]; I^2^ = 68%, *P* < 0.01) at 24-h after drug intervention, respectively. There was no significant difference between levosimendan and dobutamine on all-cause mortality in ICU (fixed effect, OR = 0.72 [0.39,1.33]; I^2^ = 0%, *P* = 0.99). We combine effect sizes related to different measurement parameters to evaluate cardiac function, which implied that septic patients with myocardial dysfunction might have a better improvement of cardiac function by levosimendan than dobutamine (random effects, SMD = 1.05 [0.69,1.41]; I^2^ = 67%, *P* < 0.01). This study suggested a significant improvement of CI, LVSWI, and decrease of blood lactate in septic patients with myocardial dysfunction in ICU after 24-h administration of levosimendan than dobutamine. However, the administration of levosimendan has neither an impact on mortality nor LVEF. Septic patients with myocardial dysfunction may partly benefit from levosimendan than dobutamine, mainly embodied in cardiac function improvement.

## Introduction

Sepsis-induced cardiac dysfunction, or sepsis-induced cardiomyopathy (SICM), is characterized by acute and reversible myocardial depression and consequent circulatory abnormalities, which are the most common clinical manifestations in septic patients^1^. With the advancement of point of care technology, from the first reported cardiac evaluation by radionuclide cineangiography in the 1980s to the most widely used bedside ultrasound evaluation in recent years^2–4^, sepsis-induced cardiac dysfunction has been found so widespread in patients with sepsis, which was largely underestimated due to technical limitations^5^. Although it is difficult to quantify which extent septic cardiomyopathy independently affects the prognosis of septic patients due to the interaction of various complex physiological and pathological variables, Vieillard-Baron and his colleagues have elegantly confirmed that septic patients with left ventricular systolic dysfunction have a higher mortality rate than patients well resuscitated without cardiac dysfunction^6^.

Inotropic agents are important therapeutic options for SICM, which are used to increase the force of cardiac contractions and improve hemodynamics. Dobutamine, a beta-1 adrenergic agonist, mainly stimulates myocardial beta-1 adrenergic receptors, resulting in increased cardiac contractility without evoking vasoconstriction or tachycardia, has been widely used to antagonize the downregulation of β adrenergic receptor for patients with persistent cardiogenic shock in ICU. Levosimendan, another attractive inotrope for cardiogenic shock in SICM, which optimizes hemodynamics with both left ventricle (LV) and right ventricle (RV) function in a catecholamine-independent pattern to minimize oxygen demand, arrhythmia, and catecholamines resistance for sepsis via calcium sensitization^7–9^. Whether levosimendan is superior to dobutamine remains a highly contentious issue, previous studies were characterized by a wide variety of opinions on this topic^7,10,11^. While few of these studies have focused on prognosis of cardiac function and outcome in patients with sepsis-induced cardiac dysfunction. More importantly, these studies did not take changes in quantitative parameters such as ejection fraction into account with baseline level.

In this meta-analysis, we aimed to determine the effects of levosimendan, comparing to dobutamine on prognosis of cardiac function, mortality and clearance of serum lactic acid in SICM patients and provide recommendations for clinical practice.

## Material and methods

The meta-analysis of randomized controlled trials (RCTs) was performed according to the Preferred Reporting Items for Systematic Reviews and Meta-Analyses (PRISMA)^12^, and a PRISMA checklist is provided separately (Additional file 1). Complete details, including electronic search strategy, objectives, criteria for study selection, eligibility, data collection, and assessment of study quality, were registered in advance in the PROSPERO International Prospective Register of Systematic Reviews (CRD42020191017).

### Search strategy

We searched Google Scholar, PubMed, Cochrane Library, Embase for potentially eligible trials by screening titles and reviewing abstracts, with no filters or publication status or language restrictions.

Two independent investigators (W.-T. C. and D.-H. L.) conducted a systematic search for RCTs published up until 12th November 2020. Inclusion criteria were prespecified according to the PICOS (population, intervention, comparison, outcomes, and study design) approach (Table 1).

**Table 1:** PICOS approach for selecting clinical studies in the systematic search.

PICOS	Criteria
Patients	Adult individuals with sepsis
Intervention	Levosimendan
Comparison	Dobutamine
Outcomes	Primary outcome: the change (before-after comparison to the baseline) of cardiac function parameters at the time point of 24-h, including ΔCI, ΔLVEF and ΔLVSWI Secondary outcomes: all-cause mortality in ICU and Δblood lactate at the time point of 24-h
Study design	Randomized controlled trials

*CI* cardiac index; *LVEF* left ventricular ejection fractions; *LVSWI* left ventricular stroke work index; *ICU* intensive care unit.

The search strategy was as follow: (“levosimendan”[MeSH Terms] OR “levosimendan”[All Fields]) AND (“Sepsis”[MeSH Terms] OR “Sepsis”[All Fields] OR “Septic”[MeSH Terms] OR “Septic”[All Fields] OR “Bacteremia”[MeSH Terms] OR “Bacteremia”[All Fields]) AND (“myocardial”[MeSH Terms] OR “myocardial”[All Fields] OR “cardiac”[MeSH Terms] OR “cardiac”[All Fields] OR “myocardium”[MeSH Terms] OR “myocardium”[All Fields] OR “heart”[MeSH Terms] OR “heart”[All Fields]).

### Study selection criteria

Randomized trials and observational studies on the use of levosimendan in adult patients with severe sepsis and septic shock will be included if reporting our primary outcomes (cardiac function parameters at the time point of baseline and 24-h, including CI, LVEF and LVSWI) and our secondary outcomes (all-cause mortality in ICU and blood lactate at the time point of baseline and 24-h). References of the previously published meta-analyses were also examined for eligible articles.

### Data extraction

Two reviewers (Y.-L. N. and D.-H. L.) independently extracted the data from all included articles. One study did not report CI; however, we decided to include this article to calculate other new functional indexes. Data extraction was performed to capture information on study-related, participant-related, and treatment-related characteristics. Authors of studies eligible for inclusion in our review were contacted if original data were missing.

Two authors (Y.-L. N. and D.-H. L.) independently and critically evaluated the methodological quality of the included studies according to The Cochrane Collaboration approaching^13^ (applying a rating of “Low risk”, “High risk” or “Unclear risk” of bias): method of random sequence generation, allocation concealment, blinding of the participants and personnel, blinding of outcome assessment, missing data reporting, selective reporting and any other kind of bias.

### Outcome measurements and definitions

We extracted data on one primary outcome and two secondary outcomes. The primary outcome was the change (before-after comparison to the baseline) of cardiac function parameters at the time point of 24-h, including ΔCI, ΔLVEF, and ΔLVSWI. Our secondary outcomes were all-cause mortality in ICU and Δblood lactate at the time point of 24-h.

### Assessment of risk of bias

Modified Jadad scale (Table 2) was used to assess the quality of evidence from the included studies (1–3 for low quality and 4–7 for high quality): random sequence production (adequate, unclear, inadequate), allocation concealment (adequate, unclear, inadequate), blinding method (adequate, unclear, inadequate), withdrawal (described, undescribed). Differences in judgment were resolved by group discussion.

**Table 2:** Modified Jadad scale.

Author	Year	random sequence generation (selection bias)	allocation concealment (selection bias)	blinding of the participants and personnel (performance bias )	blinding of outcome assessment (detection bias)	missing data reporting (attrition bias)	selective reporting (reporting bias)	other bias
Morelli^7^	2005	1	1	2	2	2	1	2
Morelli^14^	2010	1	1	2	2	2	1	2
Fang^15^	2014	2	0	0	1	2	1	2
Meng^16^	2016	2	1	1	1	2	1	2
Hajjej^17^	2017	1	1	2	2	2	1	2
Xu^18^	2018	2	1	0	0	2	1	1

Low risk = 2, unclear risk = 1, high risk = 0.

### Statistical analysis

If data was presented as median [25th;75thpercentile], the mean and standard deviation (SD) were estimated by median and quartile spacing by the corresponding formula, and the change of mean and SD from baseline after 24-h treatment is also calculated, according to the formula provided by Cochrane Handbook for Systematic Reviews of Interventions Version 6.1^19^.

**Figure 1:**
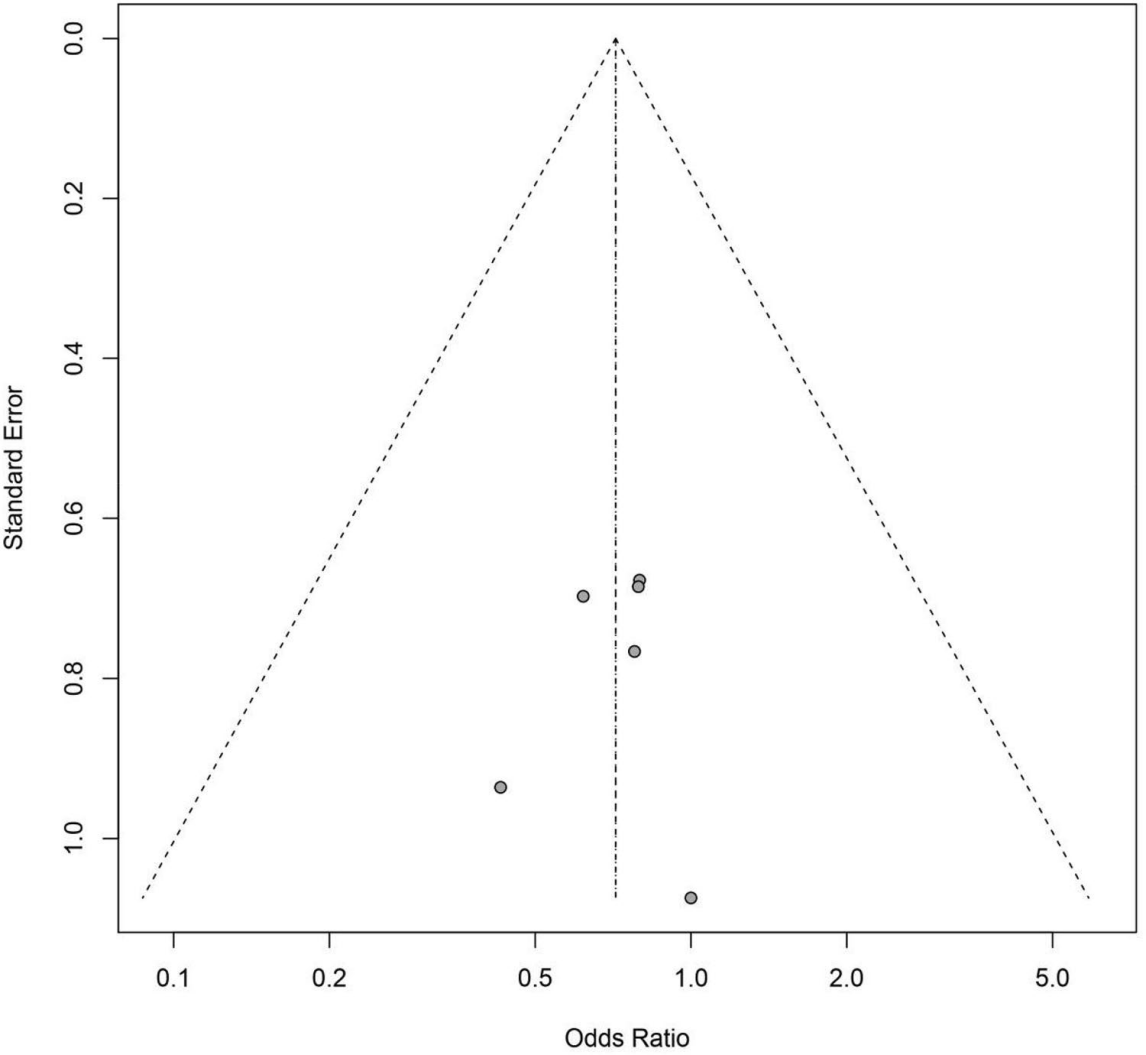
Funnel plot.

The data was analyzed as recommended in the Cochrane Handbook for Systematic Reviews of Interventions^20^. For dichotomous variables^21^, the inverse variance weighting was used, and risk ratios (RRs) with 95% confidence intervals (CIs) were calculated. Continuous outcomes^22^ were pooled through the inverse variance method and DerSimonian-Laird estimator, with the inverse variance method for random effects model and the DerSimonian-Laird estimator for fixed effects model, and we calculated the mean difference (MD) or standardized mean difference (SMD) with 95% CIs. To assess the between-trial heterogeneity, the I^2^ was applied^19,23^. Heterogeneity was judged accordingly: 0–40% = low, 30–60% = moderate, 50–90% = substantial (or high) and 75–100% = considerable. The importance of this measure depends on the magnitude and direction of effects as well as the precision of the estimate (often judged by the corresponding *P* value from the chi-squared test)^19^. Point estimates (OR), together with their corresponding 95% confidence intervals (CIs), were presented as forest plots. The presence of publication bias was assessed by funnel plot (Fig. 1)^24^. All analyses were performed with R (version 4.0.3) and meta package^25^.

### Ethics declarations

This article does not contain any studies with human participants or animals performed by any of the authors.

## Results

### Literature search

**Figure 2:**
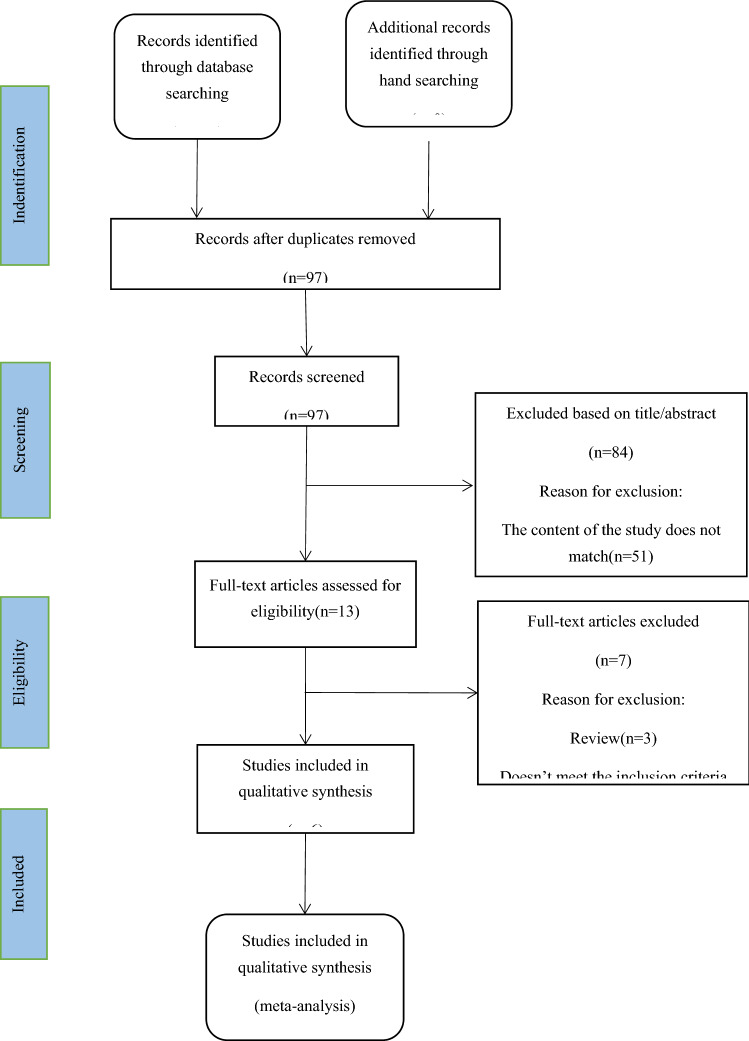
Flowchart of included studies.

We screened 358 article titles and abstracts from the electronic databases and removed 261 duplicates (Fig. 2). Due to a lack of relevant information about our predefined outcome parameters, only 13 articles were retrieved for full-text assessment. Finally, the remaining 6 studies^7,14–18^ were included in our quantitative analysis.

### Study characteristics

We included 6 studies involving 192 patients, including 97 patients in the experimental group and 95 patients in the control group. The minimum sample size was 10, and the maximum sample size was 20. Table 3 showed the detailed characteristics and main conclusions of all studies. The RCTs were published between 2005 and 2018. Five of these trials reported CI, four reported LVEF, four reported LVSWI, and all of these studies recorded blood lactate level and all-cause mortality.

**Table 3:** Characteristics of the studies in meta-analysis.

Study	Year	Country	Journal	Study type	Level of evidence	Sample size(E/C) *	Gender (M/F)*	Median age (E/C)	Intervention(E)	Intervention(C)
Morelli^7^	2005	Rome	Intensive Care Med	RCT	I	15/13	21/7	62.4 /61.5	Levosimendan	Dobutamine
Morelli^14^	2010	Rome	BioMed Central	RCT	I	20/20	30/10	68.0/66.0	Levosimendan	Dobutamine
Fang^15^	2014	China	Chin Crit Care Med	RCT	I	18/18	27/9	61.4/61.7	Levosimendan	Dobutamine
Meng^16^	2016	China	Med Sci Monit	RCT	I	19/19	24/14	55.4/50.2	Levosimendan	Dobutamine
Hajjej^17^	2017	Tunis	Shock	RCT	I	10/10	17/3	51.0/ 61.0	Levosimendan	Dobutamine
Xu^18^	2018	China	Chin J Intern Med	RCT	I	15/15	16/14	87.9/88.1	Levosimendan	Dobutamine

*E* experimental group; *C* controlled group; *RCT* randomized controlled trials.

### Cardiac function

**Figure 3:**
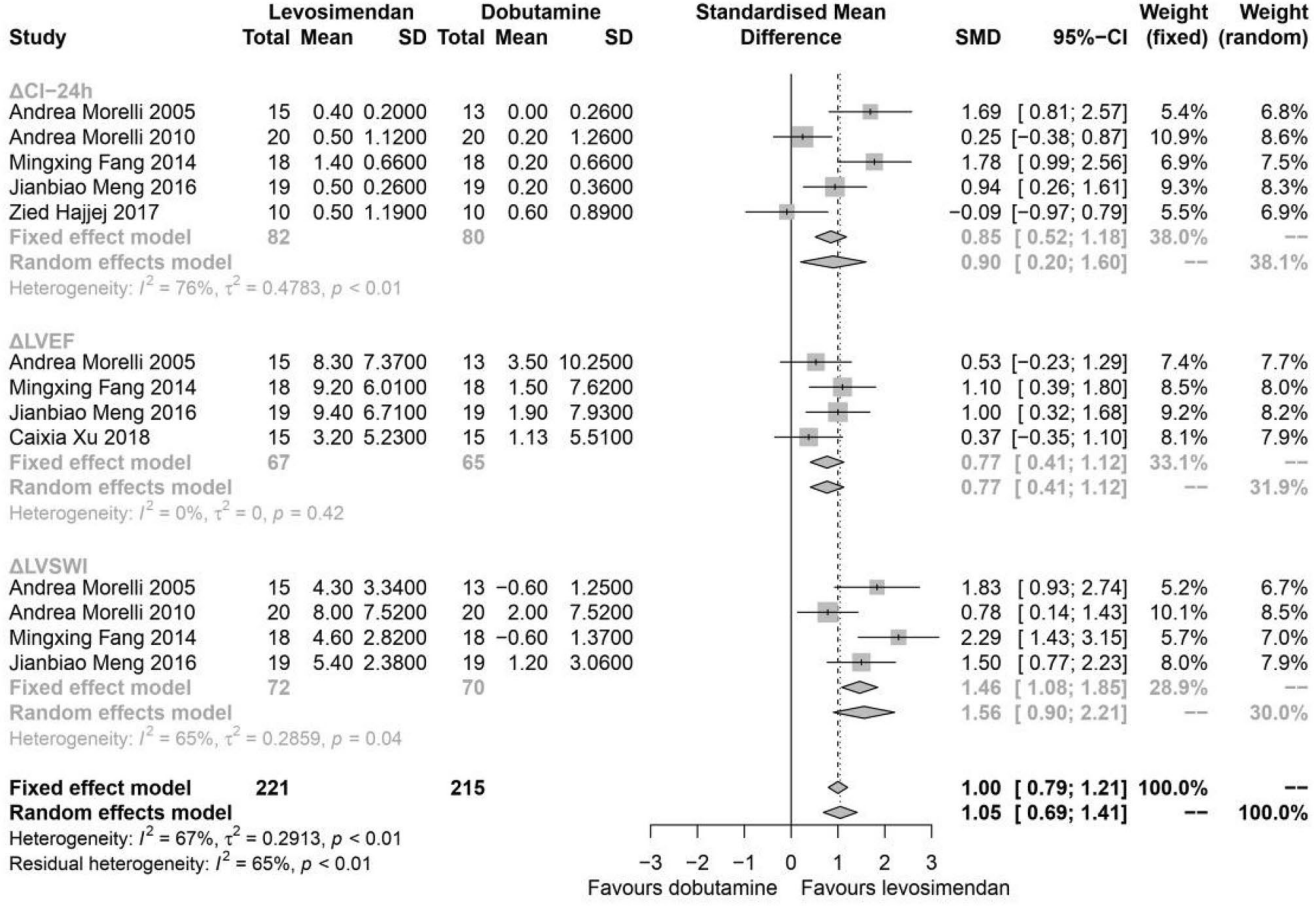
Change (before-after comparison to the baseline) of cardiac function at the time point of 24-h. ΔCI-24 h: the change (before-after comparison to the baseline) of cardiac index at the time point of 24-h; ΔLVEF: the change (before-after comparison to the baseline) of left ventricular ejection fractions at the time point of 24-h; ΔLVSWI: the change (before-after comparison to the baseline) of left ventricular stroke work index at the time point of 24-h; *SD* standard deviation; *MD* mean difference; *CI* confidence interval.

For the primary outcome, the change of cardiac function parameters including ΔCI, ΔLVEF, and ΔLVSWI at 24-h after the administration of levosimendan or dobutamine from five studies, a total of 162 patients were extracted (Fig. 3). We identified effects of levosimendan compared to dobutamine by ΔCI (I^2^ = 76%, *P* < 0.01, random effects, SMD: 0.9, 95% CIs: [0.20, 1.60]), ΔLVEF (I^2^ = 0%, *P* = 0.42, random effects, SMD: 0.77, 95% CIs: [0.41, 1.12]) and ΔLVSWI (I^2^ = 65%, *P* = 0.04, random effects, SMD: 1.56, 95% CIs: [0.9, 2.21]). In general, the change of cardiac function at 24-h was better in patients treated with levosimendan (I^2^ = 67%, *P* < 0.01, random effects, SMD: 1.05, 95% CIs: [0.69, 1.41]), subgroup analysis suggested that levosimendan improved several cardiac function parameters including CI and LVSWI.

### Clearance of serum lactic acid

**Figure 4:**
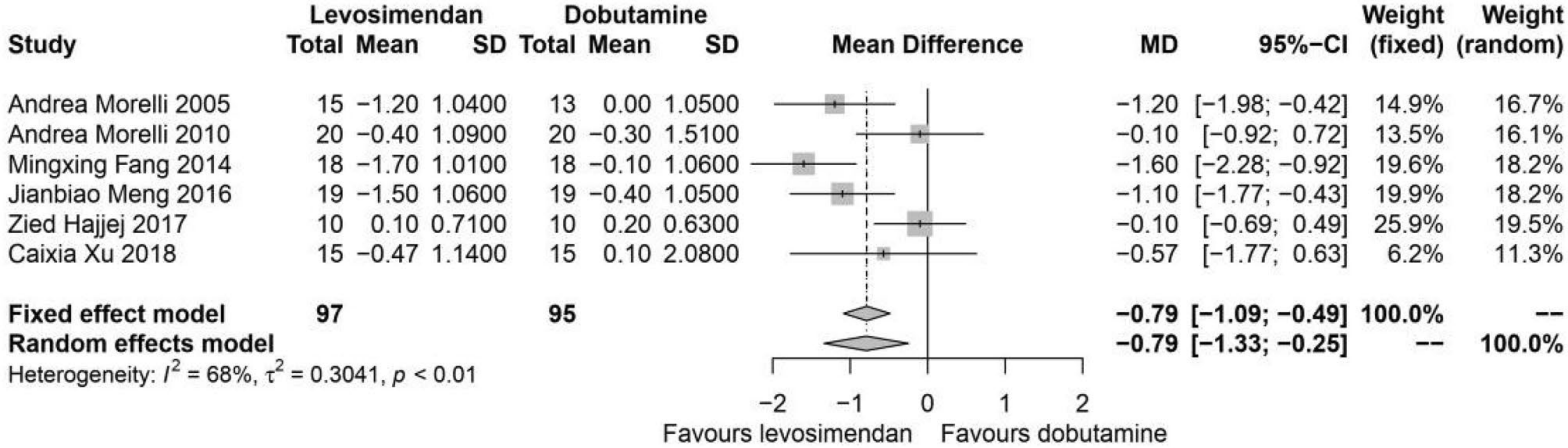
Clearance of serum lactic acid. *SD* standard deviation; *MD* mean difference; *CI* confidence interval.

All the included studies reported serum lactic acid at the time point of baseline and 24-h. Compared with dobutamine, levosimendan showed a beneficial effect on the clearance of serum lactic acid (I^2^ = 68%, *P* < 0.01, random effects, MD: − 0.79, 95% CIs: [− 1.33, − 0.25]) (Fig. 4).

### ICU all-cause mortality

**Figure 5:**
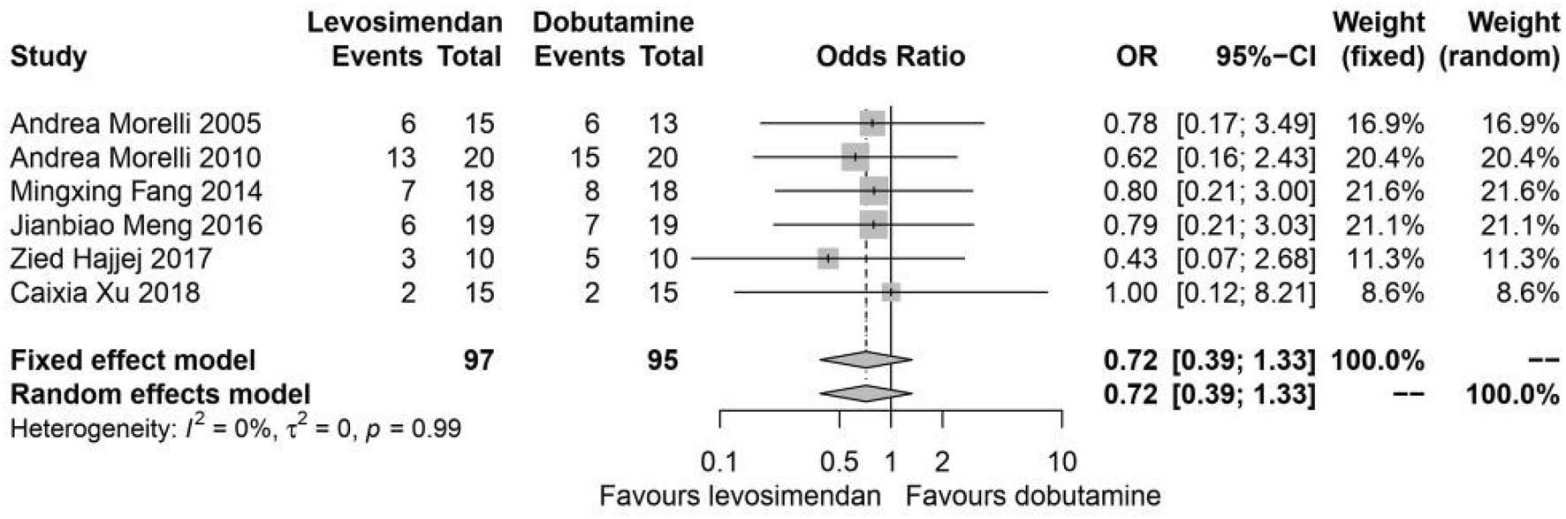
Mortality. *OR* odds ratio; *CI* confidence interval.

All the included studies reported survival status in ICU. Compared with dobutamine, levosimendan showed no beneficial effect on all-cause mortality (I^2^ = 0%, *P* = 0.99, fixed effects, OR: 0.72, 95% CIs: [0.39, 1.33]) (Fig. 5).

### Sensitivity analysis

**Figure 6:**
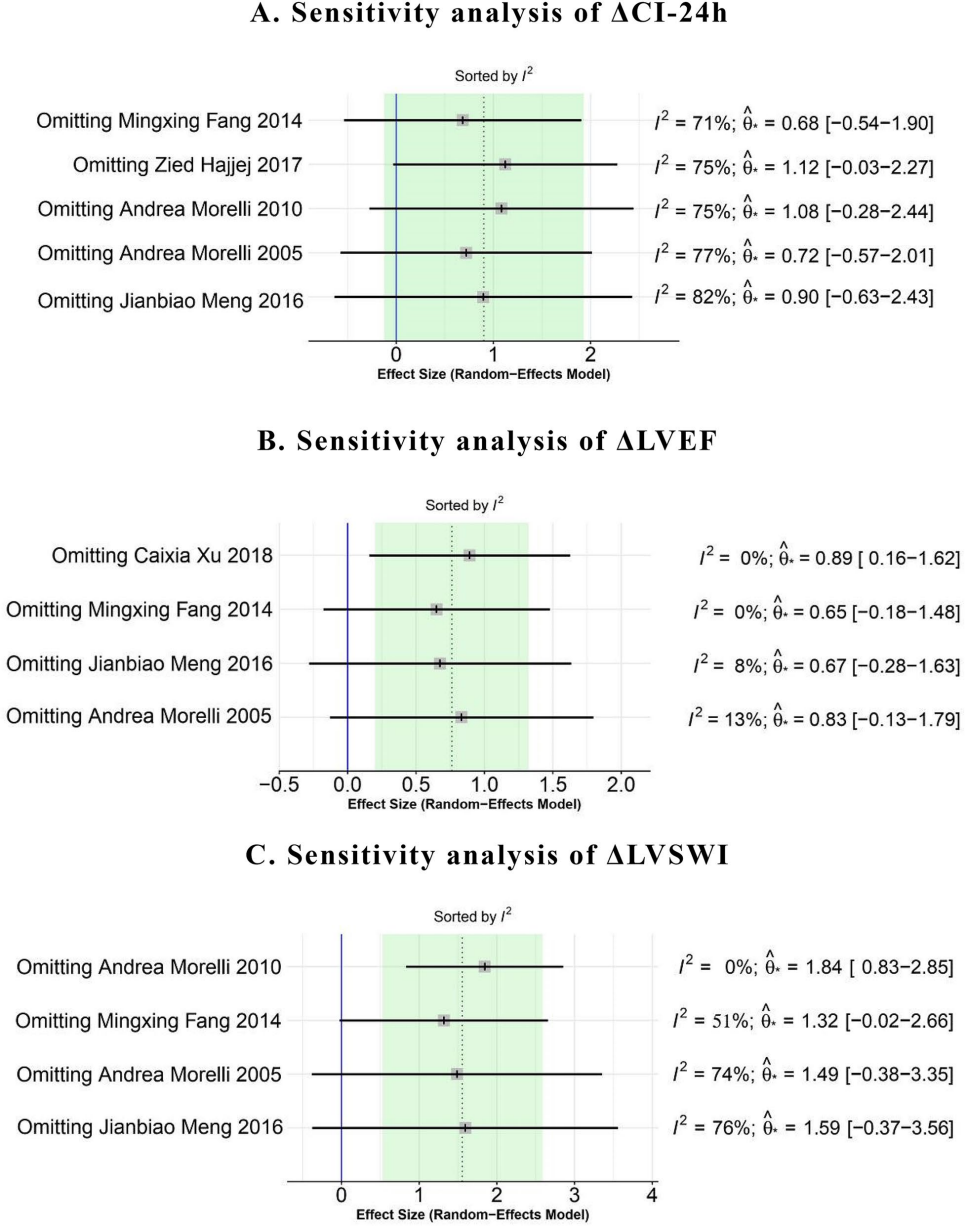
Sensitivity analysis of the changes of cardiac function parameters. (**A**) Sensitivity analysis of ΔCI-24 h. (**B**) Sensitivity analysis of ΔLVEF. (**C**) Sensitivity analysis of ΔLVSWI.

**Figure 7:**
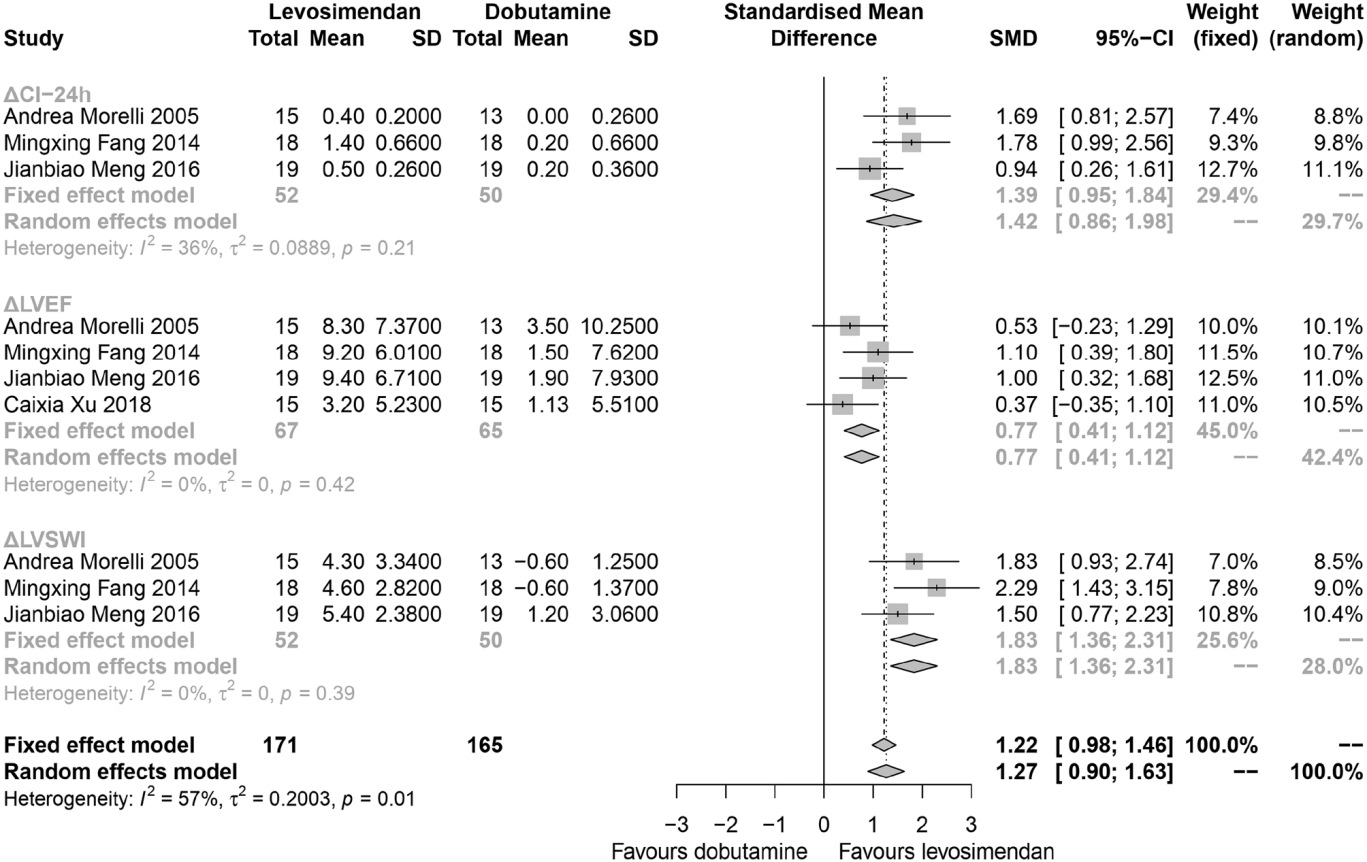
Change of cardiac function at the time point of 24-h after removing the studies from the corresponding groups. ΔCI-24 h: the change (before-after comparison to the baseline) of cardiac index at the time point of 24-h; ΔLVEF: the change (before-after comparison to the baseline) of left ventricular ejection fractions at the time point of 24-h; ΔLVSWI: the change (before-after comparison to the baseline) of left ventricular stroke work index at the time point of 24-h; *SD* standard deviation; *MD* mean difference; *CI* confidence interval.

The heterogeneity mainly existed in the result of the changes of cardiac function parameters; therefore, the sensitivity analysis was only conducted for this part (Fig. 6). The results showed that when removing morelli 2010^14^ and Hajjej 2017^17^, the heterogeneity decreased significantly in the result of ΔCI at 24-h. And in the result of ΔLVSWI at 24-h, Morelli 2010^14^ has a significant impact on the heterogeneity. After removing the studies from the corresponding groups, the fixed-effect model was used to pool the effect sizes (Fig. 7). The results showed that levosimendan could improve cardiac function to a certain extent. Yet, the heterogeneity still existed, which might be due to the different measurement methods of CI, LVSWI and LVEF.

## Discussion

To our knowledge, this is the first meta-analysis to summarize the current evidence of changes in cardiac function of SICM patients at the time point of 24-h after the administration of levosimendan (before-after comparison to the baseline). The meta-analysis results of the available data showed that levosimendan might have a significant improvement of CI and decrease of blood lactate in septic patients with myocardial dysfunction in ICU after 24-h administration of levosimendan than dobutamine.

International guidelines (2016) recommend a trial of dobutamine in the case of tissue hypoperfusion or myocardial dysfunction^26^. Dobutamine can improve myocardial contractility in patients with septic shock by exciting the myocardial beta-receptor^27,28^. Although it has also been found that dobutamine can improve the microcirculation and peripheral tissue^29^, while some clinical trials suggested that dobutamine cannot improve the outcome of septic shock patients, and even increase the mortality of 90 days^30,31^. Levosimendan, as a calcium sensitizer, is another attractive inotrope for cardiogenic shock in SICM. Unlike other inotropic agents, the positive inotropic effect of levosimendan is independent of the production of cyclic adenosine monophosphate (cAMP)^32,33^, so it could minimize oxygen demand, arrhythmia, and catecholamines resistance^7–9^. This property is of great significance for myocardial inhibition in septic patients under the hyperdynamic metabolic state. In addition, levosimendan could improve ATP-dependent potassium channels^34^: on the one hand, it could improve mitochondrial calcium overload, preserve high-energy phosphates, regulate the mitochondrial number, and exert relevant protective effects in ischemic myocardium^35^; on the other hand, levosimendan can open potassium channels of smooth muscle and regulate intracellular Ca^2+^ concentration, resulting in vasodilation and decreased peripheral vascular resistance^36^. This characteristic is of great significance for myocardial depression in septic shock with high dynamic metabolism. Decreased peripheral vascular resistance is one of the hemodynamic characteristics in septic shock and reduces left ventricular afterload^37^.

In animal experiments and clinical studies, levosimendan can lead to low blood pressure secondary to decreased peripheral vascular resistance, which correlates with its loading doses^38^. Instead of using a loading dose, a continuous intravenous infusion dose of 0.2 ug/kg/min of levosimendan was applied in all the included studies to maintain effective concentrations of vasoactive drugs. At the same time, patients in levosimendan group received more fluid and had more urinary output than patients in dobutamine group (Table 4) and there was no significant difference of the use of norepinephrine at baseline and after 24 h between the experimental and the control group. Even though fluid input was different between two groups, septic shock patients were randomized to receive either levosimendan (0.2ug/kg/min) or dobutamine (5ug/kg/min) after achieving normovolemia and a mean arterial pressure of at least 65 mmHg in all the included studies. Therefore, the improvement of left ventricular systolic dysfunction induced by levosimendan may be attributed to the fact that levosimendan could improve LV ejection capacity^39^.

**Table 4:** Some relevant data between the experimental and the controlled group in the included literature.

		Dosage regimen design	Dose of norepinephrine at baseline (ug/kg/min)	Dose of Norepinephrine at 24 h (ug/kg/min)	Fluid input (ml)	Urinary output (ml)
Morelli ^7^2005	Experimental Group	Levosimendan (0.2 μg/kg/min) for 24 h	0.22 ± 0.07	0.22 ± 0.06	5907 ± 330	2028 ± 461
	Controlled Group	Dobutamine (5 μg/kg/min) for 24 h	0.22 ± 0.05	0.23 ± 0.06	4311 ± 136	1521 ± 302
	*P* value		NA	NA	< 0.05	< 0.05
Morelli ^14^2010	Experimental Group	Levosimendan (0.2 μg/kg/min) for 24 h	0.4 (0.2–0.9)	0.3 ( 0.1–0.9 )	NA	NA
	Controlled Group	Dobutamine (5 μg/kg/min) for 24 h	0.4 (0.3–0.7)	0.4 ( 0.3–1.1 )	NA	NA
	*P* value		0.72	0.10	NA	NA
Fang^15^ 2014	Experimental Group	Levosimendan (0.2 μg/kg/min) for 24 h after dobutamine (5 μg/kg/min) for 48 h	NA	0.33 ± 0.06	5746.6 ± 420.0	2213.4 ± 354.0
	Controlled Group	Dobutamine (5 μg/kg/min) for 48 h	NA	0.33 ± 0.05	4156.7 ± 215.0	1533.8 ± 402.0
	*P* value		NA	0.909	0.000	0.000
Meng^16^ 2016	Experimental Group	Levosimendan (0.2 μg/kg/min) for 24 h	0.42 ± 0.13	0.36 ± 0.11	NA	NA
	Controlled Group	Dobutamine (5 μg/kg/min) for 24 h	0.40 ± 0.11	0.37 ± 0.09	NA	NA
	*P* value		0.619	0.761	NA	NA
Hajjej^17^ 2017	Experimental Group	Levosimendan (0.2 μg/kg/min) for 24 h	0.3 (0.1–0.8)	0.34 (0.2–0.9)	997 (842–1200)	NA
	Controlled Group	Dobutamine (5 μg/kg/min) for 24 h	0.2 (0.1–0.7)	0.27 (0.1–0.6)	898 (778–1120)	NA
	*P* value		NA	NA	-	NA
Xu^18^ 2018	Experimental Group	Levosimendan (0.2 μg/kg/min) for 24 h	23.3 ± 3.6	NA	2741 (2499–4144)	985 (530–1740)
	Controlled Group	dobutamine (5 μg/kg/min) for 24 h	23.9 ± 7.4	NA	2740 (2524–3050)	1720 (1195–2400)
	*P* value		0.591	NA	NA	NA

*NA* Not available in the included literature.

At the same time, patients were given an adequate fluid input and thus not presented with a fall in blood pressure. Although patients in levosimendan group received more fluid than patients in dobutamine group, there was no significant difference in both urine volume and EVLWI measured via PiCCO device, which may be explained by the fact that levosimendan could improve LV ejection capacity and reduce venous pressure. Previous studies have suggested that dobutamine improves cardiac contractility in patients with septic shock and that its use combined with other vasoactive agents has the potential to improve MAP^40^. Nevertheless, the present study did not show that dobutamine was superior to levosimendan in improving the CI index, probably due to beta-receptor down regulation in septic shock^41^. Therefore, low dose of 5ug/kg/min of dobutamine commonly used in other diseases may not be effective in patients with septic shock. Moreover, there is also an increased risk of arrhythmias if high doses of dobutamine are used.

Although the clearance of serum lactic acid in the levosimendan group increased significantly at 24-h after administration than in the dobutamine group, it could not be suggested that levosimendan could improve tissue perfusion due to more fluid input in levosimendan group at 24-h after administration. The effect of fluid resuscitation for tissue perfusion and organ protection in patients with septic shock is definite, and decreased lactate concentrations can not yet be deduced by the direct effect of levosimendan. Although lactate clearance could reflect microcirculation to a certain extent, it is far less intuitive than Sidestream Dark Field (SDF) imaging, a new way for clinical observation of microcirculation. In this modality, a light guide imaging the microcirculation is surrounded by light-emitting diodes of a wavelength (530 nm) absorbed by erythrocyte hemoglobin to be clearly observed as flowing cells. This method of observing microcirculation provides a clear image of capillary without blurring^42^. The development of new imaging methods, such as SDF, is more helpful to determine the critical role of treatments in improving microcirculation in sepsis.

Both levosimendan and dobutamine could improve LVEF. But according to the results of this study, there was no significant difference in the improvement of LVEF in the levosimendan group compared with the dobutamine group. The evaluation of left ventricular (LV) systolic function is of great significance for the evaluation and treatment of patients with heart disease. LVEF measured by echocardiography is one of the most commonly used indications^43^. Although LVEF was increased in both levosimendan and dobutamine groups and the delta was higher in the levosimendan group than dobutamine, the forest plot suggested that the data about ΔLVEF were heterogeneous. Previous studies had suggested that poor agreement were revealed among different methods measuring LVEF and the Simpson method had a more predictivity than the Teichholz method in evaluating LV function^44^. However, only three of the included studies clearly stated that LVEF was measured by the Simpson method (see Fang 2014^15^, Meng 2016^16^, and Xu 2018^18^) and other included literature did not. Ejection fraction calculated by Teichholz method with M-mode echocardiography from the parasternal long axis or short axis^44^, which are more susceptible to limited patient mobility and possibly mechanical ventilation^45^. It is difficult for even skilled echocardiographers to image in ICU settings. If the Teichholz method was used to measure EF in the other studies, it might increase the heterogeneity resulting in false negative result. Moreover, all parameters for evaluating LV function are affected by loading conditions that must be considered when interpreting. As early as 20 years ago, Robotham and colleagues had found that LVEF measured by an echocardiograph was a reflection of LV contractility and LV afterload^46^, especially more than a reflection of the status of LV afterload because septic cardiomyopathy was constant^47^. In 1983, Sunagawa and Sagawa proposed ventriculo-arterial coupling (VAC), the ratio of ventricular elastance to arterial elastance, which is a reliable method to quantify the cardiovascular performance and mechanical interaction between the LV and the arterial system^48^. When a VAC occurs in septic shock patients, cardiac energetics are unfavorable and usually sacrificed to maintain tissue perfusion^49^. Levosimendan had been proved to significantly improve VAC in ischemic cardiomyopathy in adults^50^ and low cardiac output syndrome in infants^51^, but whether it plays a role in patients with septic shock remains unknown and still needs clinical trials to be proved.

There had been a controversy about using levosimendan in patients with severe sepsis and septic shock due to different meta-analysis results^11,52–56^ on reducing mortality in septic individuals. Our results suggested that the administration of levosimendan had no effect on mortality and did not improve septic patients’ outcomes. Though levosimendan has no effect on mortality, at least, it can be suggested that levosimendan is superior to dobutamine in enhancing hemodynamics in a short-term effect.

The limitations of this study are as follows: Firstly, the findings and interpretations of this meta-analysis are limited by the quality of available evidence. Secondly, there is clinical heterogeneity existing in the included studies. Critically ill patients suffer from disorders other than myocardial dysfunction, such as respiratory dysfunction and neurological diseases. And considering severe sepsis and septic shock, part of the same entity could have led to heterogeneity. While interpreting our results, these confounding should be considered carefully. Thirdly, the length of follow-up for mortality was not identical among trials, and we decided to use the longest follow-up reported. Four studies reported intensive care unit mortality^7,14,15,17^, two reported 28-day mortality^16,18^. Furthermore, not every outcome of interest was recorded in each of our included studies, and insufficient data hindered comprehensive analysis. Therefore, more high-quality RCTs should be conducted to provide reasonable and firm evidence for patients.

## Conclusion

This study suggested a significant improvement of CI, LVSWI, and decrease of blood lactate in septic patients with myocardial dysfunction in ICU after 24-h administration of levosimendan than dobutamine. However, the administration of levosimendan has neither an impact on mortality nor LVEF. Septic patients with myocardial dysfunction may partly benefit from levosimendan than dobutamine, mainly embodied in the improvement of cardiac function. Further studies are needed in the future to fully clarify the effectiveness of levosimendan and dobutamine during the therapy of cardiac dysfunction induced by sepsis in ICU.

## Supplementary Information

Supplementary Information.

## Data Availability

Because this is a meta-analysis, all of data included in this study could be found in the included references.
